# Imidazolium Ionic Liquid Functionalized Carbon Nanotubes for Improved Interfacial Charge Transfer and Simultaneous Determination of Dihydroxybenzene Isomers

**DOI:** 10.3390/molecules21050617

**Published:** 2016-05-14

**Authors:** Huan Wei, Xiao-Shuai Wu, Guo-Yun Wen, Yan Qiao

**Affiliations:** 1 Chongqing Key Laboratory for Advanced Materials & Technologies of Clean Energies, Southwest University, Chongqing 400715, China; weihuannwnu@163.com (H.W.); wuxiaoshuai@163.com (X.-S.W.); wenguoyun1027@126.com (G.-Y.W.); 2 Faculty of Materials & Energy, Southwest University, Chongqing 400715, China

**Keywords:** carbon nanotube, ionic liquids, dihydroxybenzene isomers, simultaneous determination

## Abstract

In this paper; an imidazolium ionic liquid (IL) is used to functionalize multi-walled carbon nanotubes (MWNTs) by covalent bonding on the MWNT surface. The functionalization not only provides a hydrophilic surface for ion accessibility but also prevents the aggregation of MWNTs. The IL-functionalized MWNTs were then applied for the electrochemical determination of the dihydroxybenzene isomers hydroquinone (HQ); catechol (CC); and resorcinol (RC), exhibiting excellent recognition ability towards the three compounds. The linear calibration ranges for HQ; CC and RC are 0.9–150 μM; 0.9–150 μM and 1.9–145 μM and the detection limits are found to be 0.15 μM for HQ; 0.10 μM for CC and 0.38 μM for RC based on S/N of 3. The proposed electrochemical sensor was also found to be useful for the determination of the dihydroxybenzene isomers in Yellow River water with reliable recovery.

## 1. Introduction

Multi-walled carbon nanotubes (MWNTs) are important nanomaterials for fuel cells, super capacitors and electrochemical sensors because of their superior electrocatalytic performance that benefits from their fast interfacial electron-transfer reactions. However, the aggregation of MWNTs, even for some functionalized ones, is a major problem when they are used in electrochemical devices. It is thus necessary to develop an appropriate functionalization strategy that can prevent the aggregation of the MWNTs but at the same time not affect their electrocatalytic properties. Room temperature ionic liquids have quite extensive applications in electrochemical devices due to their unique electrochemical properties such as wide electrochemical windows, good biocompatibility, high conductivity, good dissolving capability and thermal stability [1]. IL-modified CNTs exhibit switchable solubility, high charge-transfer activity and high electronic conductivity that can be attributed to their inherent electrocatalytic activity.

Dihydroxybenzene isomers, namely catechol (CC), resorcinol (RC) and hydroquinone (HQ), are highly toxic environmental pollutants that are difficult to degrade [2]. These isomers usually coexist and interfere with each other during their determination due to their similar structures. Therefore, the simultaneously determination of these isomers is quite important. So far, several analytical methods have been proposed for the determination of these three compounds, including chromatography [3], high performance liquid chromatography (HPLC) [4], spectrophotometry [5] and colorimetric [6] techniques. Among all these methods, electrochemical methods are some of the most favorable because of their low cost, high sensitivity and facile operation. However, the redox peaks of these isomers (especially HQ and CC) usually overlap so that it is hard to achieve their simultaneous determination with conventional electrodes. In recent reports, this was achieved by using a single-walled carbon nanohorn modified glassy carbon electrode [7], a Nafion/multi-walled carbon nanotubes/carbon dots/multi-walled carbon nanotubes-modified glassy carbon electrode [8] or cyclodextrin-functionalized hollow carbon nanospheres [9], *etc.* Although the sensitivity of these systems is good, the electrode fabrication is too complicated, so there is a need to develop novel electrodes with facile manufacturing processes and low cost but high sensitivity and low detection limit for dihydroxybenzene isomers.

In this work, imidazolium ionic liquid covalent bonded MWNTs (MWNTs-IL) were developed with a facile synthetic procedure and used for dihydroxybenzene isomer determination. The sensitivity and detection limit were evaluated via cyclic voltammetry (CV) and differential pulse voltammetry (DPV) methods. The mechanism of the superior electrocatalytic properties of the MWNTs-IL was discussed and the developed sensor was also applied to the simultaneous determination of the dihydroxybenzene isomers in Yellow River water samples.

## 2. Results and Discussion

### 2.1. Structural Properties of MWNTs and MWNTs-IL Composite

**Figure 1:**
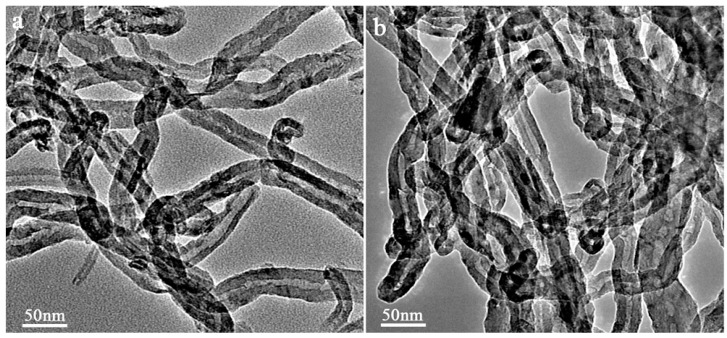
TEM micrographs of MWNTs (**a**) and MWNTs-IL composite (**b**).

TEM micrographs (Figure 1) show that the IL modification does not change the morphology of the MWNTs a lot. It suggests that a thin layer of ILs deposited on the surface of the MWNTs. It is interesting that there are more open ends (the dark O-rings in Figure 1b) in the MWNTs-IL than in the MWNTs, which suggests that the MWNTs were shortened during the IL modification process. This will decrease the possibility of tangling of the nanotubes.

**Figure 2:**
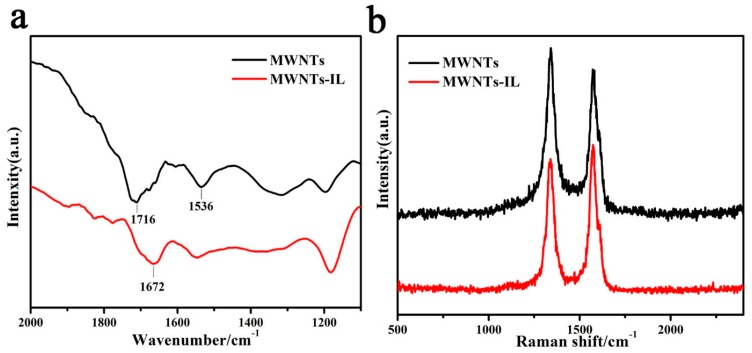
FTIR (**a**) and Raman spectra (**b**) of MWNTs and MWNTs-IL composite.

The FTIR spectra of plain MWNTs and MWNTs-IL composite are shown in Figure 2a. The C=O bands appearing at 1716 and 1536 cm^−1^ indicate the presence of carboxylic acid groups on the surface of the MWNTs [10]. For the MWNTs-IL composites, the carboxylic acid group C=O band at 1716 cm^−1^ is shifted to 1672 cm^−1^, suggesting the formation of amide bonds [11]. In addition, the assembled films of plain MWNTs and MWNTs-IL composite were also characterized by Raman spectroscopy (Figure 2b). The D band corresponds to defects in the curved graphene sheet, tube ends and staging disorder, while the G band is related to the graphitic hexagon-pinch mode [12]. The peaks at 1339 cm^−1^ and 1574 cm^−1^ can be attributed to the D-band and G-band from the MWNTs [13] and MWNTs-IL, and the R values (R = D/G) are 1.10 and 0.89 for MWNTs and MWNTs-IL, respectively. These results indicate that the addition of IL on MWNTs decreases the defects in the structure of the MWNTs and also proves that the MWNTs have been successfully modified by IL-NH_2_. 

**Figure 3:**
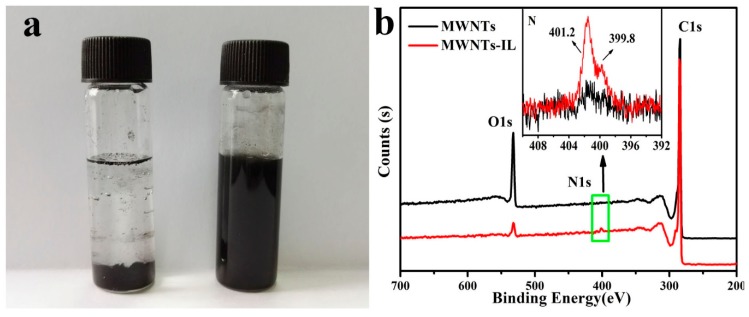
(**a**) Photographs of MWNT (**left**) and MWNT-IL (**right**) suspensions in DI water after 24 h standing; (**b**) XPS survey spectra of MWNTs and MWNTs-IL composite (inset: the N 1s count of different materials).

To evaluate the dispersion properties of the MWNT-IL composite, 1 mg of MWNTs and composite powder were put into 2 mL DI water to prepare suspensions. After fully mixing, the suspensions were left standing for 24 h to check their separation. From Figure 3a, it is obvious that the MWNTs are deposited on the bottom of the bottle but the MWNT-IL suspension still looks homogeneous. This demonstrates that the IL functionalization can greatly improve the dispersion properties of MWNTs. The reason might be that the modification with a thin IL layer provides lots of hydrophilic functional groups and the positive charge of ILs could decrease the π-π interactions between the MWNTs to avoid the tangling of MWNTs. Furthermore, the results of X-ray photoelectron spectroscopy (XPS) further support the presence of the ionic liquid with the appearance of N 1s 399.8 and 401.2 eV peaks from the imidazolium ring of the ionic liquid [14]. The qualitative XPS analyses of the MWNTs and MWNTs-IL are shown in Figure 3b. From the inset of Figure 3b, the N 1s peaks at 399.8 and 401.2 eV suggest the presence of imidazole moietiesd in the MWNTs-IL hybrids. This suggests that the aminopropylimidazole moiety in MWNTs-IL is covalently bonded onto the surface of the MWNTs, which is in agreement with the FTIR results. The weak N1s peak of MWNTs should be due to the remains from the pre-treatment of MWNTs in concentrated nitric acid. 

### 2.2. Electrochemical Characterization of MWNT-ILs Modified Electrode

**Figure 4:**
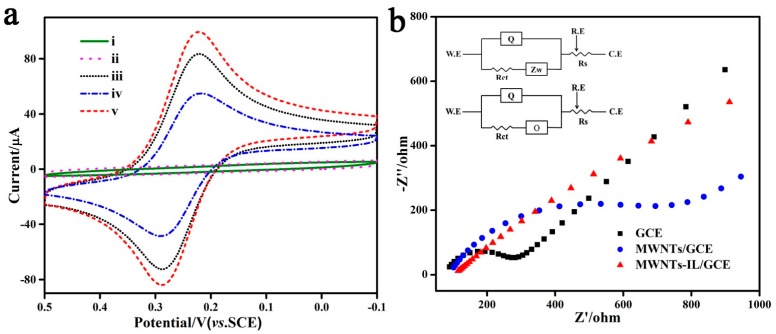
(**a**) CVs of (i) bare GCE; (ii) MWNTs-IL/GCE in 0.1 M KCl at a scan rate of 0.05 V·s^−1^; (iii) bare GCE; (iv) MWNTs/GCE; (v) MWNTs-IL/GCE in 5.0 mmol·L^−1^ K_3_Fe(CN)_6_ and 0.1 M KCl at a scan rate of 0.05 V·s^−1^; (**b**) EIS of different electrodes in a solution of 1.0 × 10^−3^ M K_3_[Fe(CN)_6_] and 0.1 M KCl. The inset is the equivalent circuit for the modified electrode in the cell.

The MWNT-ILs and MWNTs were deposited on a glassy carbon electrode (GCE) and their electrochemical behaviors were investigated via cyclic voltammetry (CV) and electrochemical impedance spectroscopy (EIS). Figure 4a shows the CV responses of bare GCE, MWNTs/GCE, and MWNTs-IL/GCE modified electrodes in K_3_Fe(CN)_6_/KCl solution. The MWNTs/GCE (curve iv) shows a decreased peak current over GCE, which might be ascribed to the inaccessibility of the electrode surface to the ferricyanide ions due to the hydrophobic surface of the MWNTs (Figure S1 in the Supplementary Materials) [15], while for MWNTs-IL/GCE, the redox peaks are greatly increased and the peak current is higher than that of the bare GCE (curve v). This suggests that the ionic liquid- functionalized MWNTs could provide a large ion-accessible surface and facilitate the interfacial charge transfer. Furthermore, electrochemical impedance spectroscopy (EIS) was also used to investigate the interfacial properties of the modified electrodes (Figure 4b). Simulated circuits were used to show the charge transfer resistance (Rct) as well as the diffusion elements. The Rct corresponding to the semi-circle in the high frequencies region should be attributed to the redox reaction of ferricyanide ions on the electrode surface. From the results, the MWNTs-IL/GCE has the smallest charge transfer resistance, which suggests that the MWNTs-IL/GCE could achieve faster interfacial electron transfer than the other two electrodes. According to the simulated EIS circuits, the diffusion models for bare the GCE and MWNTs-IL/GCE are different. The Warburg impedance (semi-infinite diffusion) element is suitable for the bare GCE while for the MWNTs-IL/GCE, it changes to an “O” element (finite diffusion) [16], suggesting the MWNTs-IL/GCE has a smaller diffusion impedance than the bare GCE.

### 2.3. Redox Behavior of Dihydroxybenzene Isomers on MWNTs-IL/GCE

**Figure 5:**
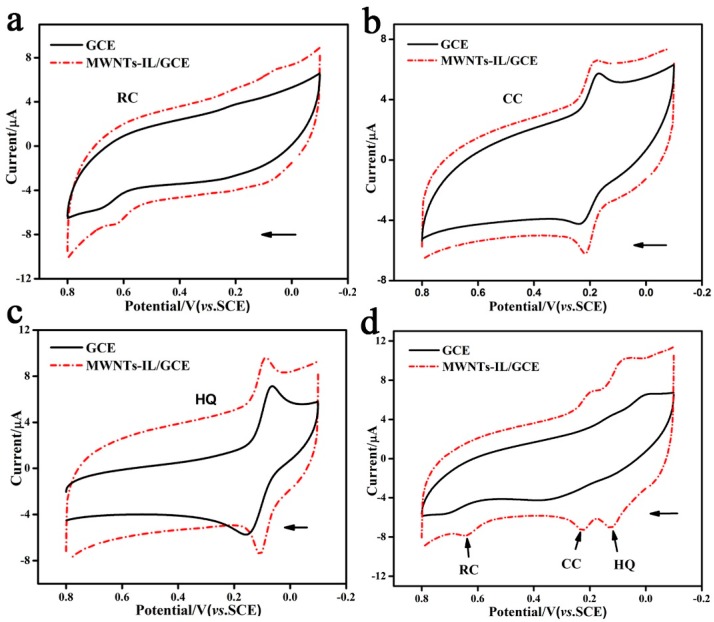
(**a**) CVs of the 50 μM RC in 0.1 M PBS at bare GCE and MWNTs-IL/GCE; (**b**) CVs of the 50 μM CC in 0.1 M PBS at bare GCE and MWNTs-IL/GCE; (**c**) CVs of the 50 μM HQ in 0.1 M PBS at bare GCE and MWNTs-IL/GCE; (**d**) the mixed components of 50 μM HQ, CC and RC in 0.1 M PBS at bare GCE and MWNTs-IL/GCE at a scan rate of 0.05 V·s^−1^.

The redox behaviors of dihydroxybenzene isomers (HQ, CC and RC) at the bare GCE and MWNTs-IL/GCE have been studied by CV. Figure 5a–c show the CV curves of 50 μM RC, CC and HQ, respectively, in 0.1 M PBS (pH 7.0) at a scan rate of 50 mV·s^−1^. For HQ and CC, the peak separations on MWNTs-IL/GCE are much smaller than that on the bare GCE. This suggests that the MWNTs-IL/GCE can achieve much faster redox reactions than the bare GCE.

The cyclic voltammograms of a bare GCE and MWNTs-IL/GCE in a mixed solution containing HQ, CC, and RC (50 μM for each) are shown in Figure 5d. For the bare GCE, the oxidation peak at 716 mV corresponds to the oxidation of RC. As the oxidation peak potentials of HQ and CC are too close, their oxidation peaks merge into one broad peak and shift to a more positive potential at around 394 mV. In this case, it is impossible to simultaneously detect HQ and CC on a bare GCE. On the other hand, for the MWNTs-IL/GCE, three well-defined oxidation peaks are observed at 125 mV, 225 mV and 641 mV, corresponding to the oxidation peaks of HQ, CC and RC, respectively, according to their CVs. This suggests that the three isomers of dihydroxybenzene can be determined simultaneously using the MWNTs-IL/GCE. It is also noted that the oxidation peak potentials of the three isomers at the MWNTs-IL/GCE are more negative than on the bare GCE. This could be explained by the improved electrocatalysis of the ionic liquid towards the oxidation of the dihydroxybenzene isomers.

### 2.4. Determination of Dihydroxybenzene Isomers at MWNTs-IL/GCE

#### 2.4.1. Individual Determination of HQ, CC or RC in Their Mixtures

**Figure 6:**
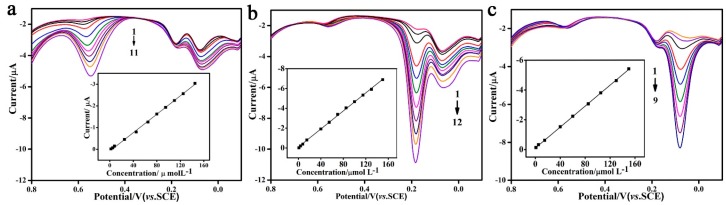
(**a**) DPV graphs of: 1.9, 5.0, 8.0, 25, 45, 65, 80, 95, 110, 125, 145 μM (from 1 to 11) RC in the presence of 20 μM HQ and CC. Insert: Linear relationship of the peak current *vs.* the concentration of RC; (**b**) DPV graphs of: 0.9, 4.0, 8.0, 15, 40, 55, 70, 85, 100, 115, 130, 150 μM (from 1 to 12) CC in the presence of 20 μM HQ and RC. Insert: Linear relationship of the peak current *vs.* the concentration of CC; (**c**) DPV graphs of: 0.9, 5.0, 15, 40, 60, 85, 105, 130, 150 μM (from 1 to 9) HQ in the presence of 20 μM CC and RC. Insert: Linear relationship of the peak current *vs.* the concentration of HQ.

As dihydroxybenzene isomers normally coexist in test samples, it is very important to eliminate the interferences of each other for the selective detection of one species. To selectively detect HQ (or CC, RC) in the presence of the other two isomers, electrochemical detection was performed on the MWNTs-IL/GCE modified electrode by DPV (Figure 6). Figure 6c displays the oxidation peak current of HQ in the presence of 20 μM CC and RC, which is linearly proportional to its concentration in the range of 9.0 × 10^−7^ M~1.5 × 10^−4^ M with a regression equation of i_p_ = 0.118–0.035c. Similar behaviors were observed for RC or CC, that are shown in Figure 6a,b. The regression equations, linear correlation coefficients, peak positions, concentration ranges and detection limits (S/N = 3) are summarized in Table 1. Thus, the above results show that the peak currents are linearly proportional to the concentrations of HQ (or CC, RC) while the peak currents of the other two isomers almost remain constant although the peaks seem to be increasing at the base line. This suggests that the oxidations of HQ, RC and CC at the MWNTs-IL/GCE modified electrode take place independently and there is no significant interference between each other. Table 2 summarizes this sensor performance based on MWNTs-IL composite and other relevant sensor materials collected from the literature. These results reveal that the method proposed in this work had higher sensitivity and lower detection limits than previously reported works, and it could be a reliable technique for the simultaneous and quantitative determination of coexisting HQ, CC and RC.

#### 2.4.2. Simultaneous Determination of Dihydroxybenzene Isomers at MWNTs-IL/GCE

**Figure 7:**
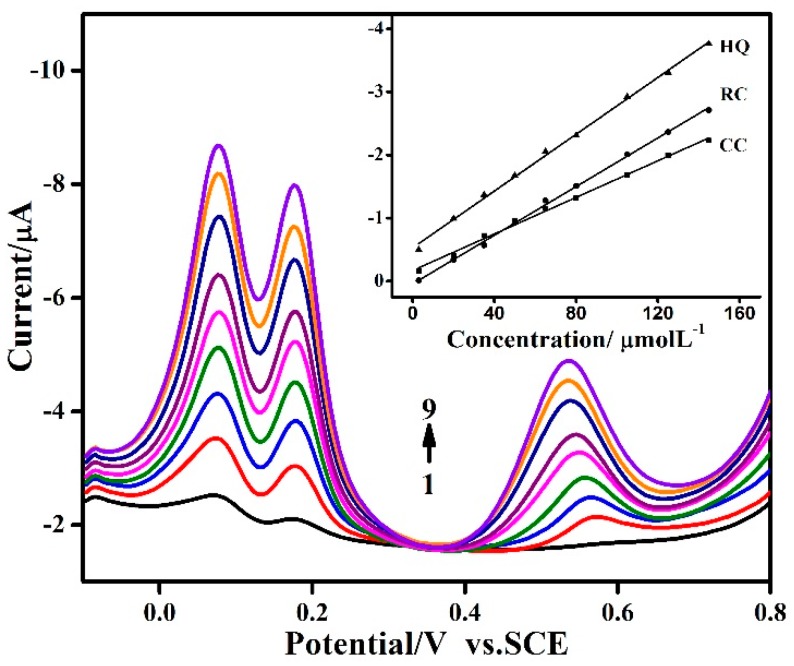
DPV graphs of MWNTs-IL/GCE in the presence of HQ, CC and RC, each concentration: 3.0, 20, 35, 50, 65, 80, 105, 125, 145 μM (from 1 to 9) in 0.1 M PBS (pH 7.0). Insert: Linear relationship of the peak currents *vs.* the concentrations of HQ, CC and RC.

The simultaneous determination of HQ, RC and CC at the MWNTs-IL/GCE modified electrode was further studied by synchronously changing the concentrations of HQ, RC and CC. As shown in Figure 7, the peak currents of HQ, CC and RC increased linearly with their individual concentration in the range of 3.0 × 10^−6^~1.45 × 10^−4^ M. This response proves further that the oxidation of the three isomers at the MWNTs-IL/GCE occurs independently. The inset of Figure 7 is the calibration plots of the peak current (i) *vs.* concentration (c). The regression equations for HQ, CC and RC are i_HQ_ = −0.530 − 0.023c (μM), i_CC_ = −0.170 − 0.014c (μM) and i_RC_ = 0.042 − 0.019c (μM), respectively. Hence, the modified electrode was quite suitable for the simultaneous detection of mixed dihydroxybenzene systems, and the method was simple, rapid and accurate. 

The reproducibility and stability of the composite electrode were evaluated by DPV. Under the optimized conditions, the MWNTs-IL/GCE was used to determine 50 μM HQ, RC and CC five times by DPV and the relative standard deviation (RSD) of the peak currents for HQ, CC and RC were 2.18%, 2.76%, 3.64%, respectively. After the electrode was stored at 4 °C for a week, the current response reached approximately 99.3% of its initial current sensitivity. These results indicate that the proposed sensor has excellent reproducibility and stability.

#### 2.4.3. Application to the Yellow River Samples

To demonstrate the analytical applicability of the proposed method, the concentrations of dihydroxybenzene isomers in Yellow River water samples were determined with the MWNTs-IL/GCE electrode, and we found that the contents of dihydroxybenzene isomers in the Yellow River water were below the detection limits. Thus, different concentrations of the dihydroxybenzenes were added to prepare the artificial wastewater and then their concentrations were evaluated and their recoveries calculated. The results are summarized in Table 3. These results indicate that it is feasible to apply the proposed method to determine dihydroxybenzene isomers in real samples.

## 3. Discussion

In a previous report, ILs were used as electrolyte for a CNT electrode [20], or just mixed with CNTs to form a gel [21]. In this work, the IL was covalently bonded with CNTs through amide groups. As the formation of amide groups depends on the number of carboxyl groups on the CNT surface, which are also the defect sites of CNTs, the bonding of ILs on these defect sites could fill up the gaps for the electron transfer so the MWNT-IL composite displayed greatly improved interfacial charge transfer rates.

For the simultaneous determination of dihydroxybenzene isomers, the problem is that the redox potential values of HQ and CC are too close. If the electrode kinetics of the interfacial redox reaction are poor, the two redox pairs will affect each other and thus be hard to divide into two peaks. The MWNT-IL/GCE electrode possesses fast interfacial charge transfer so that the redox peaks for HQ and CC could be narrower than those on GCE. This should be the reason why this MWNT-IL/GCE electrode can achieve the simultaneous determination of dihydroxybenzene isomers. It is also possible that there are interactions between dihydroxybenzene isomers and imidazolium groups that can anchor the isomers on the surface of electrode and thus decrease the diffusion distance. In the future we will further clarify the mechanism details and also investigate the effect on the determination of dihydroxybenzene isomers using ionic liquids with different anions. 

## 4. Experimental Section

### 4.1. Reagents

The multi-walled carbon nanotubes (MWNTs, diameter: 20–40 nm, length: 1–2 μm, purity: ≥ 95%) came from Shenzhen Nanotech Port Co. Ltd. (Shenzhen, China). Hydroquinone, catechol and resorcinol (Fuchen Chemical Reagent Factory, Tianjin, China), potassium ferricyanide and potassium chloride (Xi’an Chemical Reagent Factory, Xi’an, China) were of analytical reagent grade. PBS (pH 7.0) was prepared by mixing suitable amounts of 0.1 M NaH_2_PO_4_/Na_2_HPO_4_. Other chemicals were all analytical grade, and the solutions were prepared using deionized water. 

### 4.2. Preparation of MWNTs-IL

IL functionalized MWNTs (MWNTs-IL) were prepared by a previously reported method [22]. Briefly, it is based on an amidation reaction between carboxylic acid group functionalized MWNTs (MWNTs–COOH) and the amine-terminated IL (1-propylamine-3-methylimidazolium bromide, IL-NH_2_) (Figure S2). The pristine MWNTs sample was sonicated in a mixture of concentrated H_2_SO_4_ (65%)/HNO_3_ (98%, 3:1 by volume) for a few minutes followed by refluxing under magnetic stirring at 60 °C for 5 h using an oil-bath, and then washed with distilled water to neutrality and dried in vacuum at 70 °C. The IL-NH_2_ was prepared by reaction of 1-methylimidazole (0.02 mol) with 3-bromopropylamine (0.02 mol) in 50 mL ethanol under reflux in N_2_ atmosphere for 24 h, then the ethanol was removed by vacuum filtration, followed by extraction of the residue with ethanol-THF, in which the imidazolium salt is soluble. 

MWNTs-IL was prepared by ultrasonicating a solution of 5 mg of the MWNTs–COOH, 10 mg of IL-NH_2_, and 10 mg of dicyclohexylcarbodiimide (DCC) in 10 mL of dimethylformamide (DMF) for 15 min, and then vigorously stirring at 50 °C for 24 h. Then, unreacted MWNTs were removed by centrifugation. After that, MWNTs-IL was filtered through a nylon membrane with 0.22 μm pores, and thoroughly washed with DMF, ethanol and water, respectively. 

### 4.3. Electrode Preparation

Before modification, the GCE was polished with 0.05 μm alumina slurry and then cleaned ultrasonically in deionized water. Finally, the GCE was coated with 5 μL of 2 mg/mL MWNTs-IL suspension and the solvent evaporated under room temperature for 1 h. The surface morphology of MWNT-IL/GCE shows that a uniform thin layer of MWNT-IL is deposited on the GCE surface. (Figure S3) The modified electrode was cleaned with deionized water before use.

### 4.4. Instruments

Structural characterization of the studied materials was conducted using a Perkin Elmer-Fourier transform infrared (FTIR) spectroscopy instrument (Nicolet 6700 FTIR, Thermo Electronic Corporation, West Chester, PA, USA) in the range of 2000–500 cm^−1^ in KBr pellets. Raman spectra were recorded by a Renishaw 2000 system (LabRam HR 800,HORIBA Jobin Yvon, Palaiseau, France) equipped with an argon ion laser (514.5 nm) and a charge-coupled device detector. X-ray photoelectron spectroscopy (XPS) analysis was carried out on an ESCALAB MK II X-ray photoelectron spectrometer (Thermo escalab 250i, Thermo Electronic Corporation).

The electrochemical measurements were conducted in a three electrode cell system (Shanghai Chenhua Co., Shanghai, China) using a GCE (3.5 mm diameter) or a modified GCE as the working electrode, a Pt wire as the counter electrode and a saturated calomel electrode (SCE) as the reference electrode, using a CHI660C electrochemical workstation (Shanghai Chenhua Co.). Phosphate buffer solution (0.1 M, pH 7.0) with a certain amount of HQ, CC and RC was transferred into the cell as the electrolyte. All experiments were carried out at room temperature.

## 5. Conclusions

In this work, the constructed MWNTs-IL/GCE exhibits excellent recognition ability toward dihydroxybenzene isomer electro-oxidation. CV results showed that the MWNTs-IL-modified electrode could improve the interfacial charge transfer and increase the peak current. The isomers *o*-, *m*- and *p*-dihydroxybenzene can be simultaneously determined at the modified electrode. The results may be attributed to the special nanoelectrochemical interface where dihydroxybenzene isomers possess dissimilar conformations that leads to different peak potentials. The proposed method could be used for monitoring dihydroxybenzene isomer pollutants in the environment.

## Figures and Tables

**Table 1:** Linear ranges and detection limits of dihydroxybenzene isomers (*n* = 6).

Isomer	Linear Regression Equation	R	Peak/mV	Linear Range/mol·L^−1^	Detection Limit/mol·L^−1^
CC	i_p_ = −0.071–0.046c	0.999	180	9.0 × 10^−7^–1.5 × 10^−4^	1.0 × 10^−7^
RC	i_p_ = 0.060–0.021c	0.998	547	1.9 × 10^−6^–1.45 × 10^−4^	3.8 × 10^−7^
HQ	i_p_ = 0.118–0.035c	0.999	78	9.0 × 10^−7^–1.5 × 10^−4^	1.5 × 10^−7^

**Table 2:** Performance comparison of MWNTs-IL/GCE with other electrodes for HQ, CC, and RC detection.

Electrode	Linear Range (μM)			Limit of Detection (μM)			Ref.
	HQ	CC	RC	HQ	CC	RC	
SWCNH/GCE	0.5–100	0.5–100	1.0–100	0.10	0.20	0.50	[7]
MWNTs/CDs/MWNTs/GCE	1.0–200	4.0–200	1.0–400	0.60	0.60	1.00	[8]
MWCNTs/GCE	20–140	20–140	20–140	1.00	1.30	4.70	[17]
TH-MWNTs/GCE	0.9–360	3.3–810	4.3–900	0.27	1.00	1.10	[18]
CNx/GCE	10–1000	20–1000	50–1000	1.20	2.71	5.64	[19]
MWNTs-IL/GCE	0.9–150	0.9–150	1.9–145	0.15	0.10	0.38	This paper

**Table 3:** Determination of dihydroxybenzene isomers in the Yellow River Water samples (*n* = 3).

Isomer	Added (μM)	Found (μM)	Recovery (%)
CC	20.00	19.02	95.1
	25.00	24.62	98.5
RC	20.00	21.73	108.7
	25.00	25.96	103.8
HQ	20.00	21.09	105.4
	25.00	26.51	106.0

## Supplementary Materials

Supplementary materials can be accessed at: http://www.mdpi.com/1420-3049/21/5/617/s1.
