# Supplementary material for: Imidazolium Ionic Liquid Functionalized Carbon Nanotubes for Improved Interfacial Charge Transfer and Simultaneous Determination of Dihydroxybenzene Isomers
Source: Molecules. 2016 May 14;21(5):617. doi: 10.3390/molecules21050617 (PMC6274356; doi:10.3390/molecules21050617)
Supplement: Supplementary file 1 [file molecules-21-00617-s001.pdf]

Supplementary Materials: Imidazolium Ionic Liquid Functionalized Carbon Nanotubes for Improved Interfacial Charge Transfer and Simultaneous Determination of Dihydroxybenzene Isomers

Huan Wei, Xiao-Shuai Wu, Guo-Yun Wen and Yan Qiao

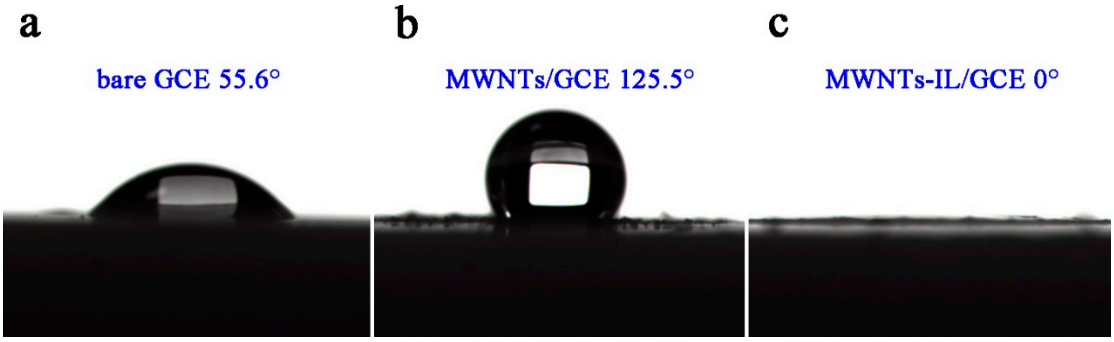

Figure S1. Water contact angle of bare GCE (**a**), MWNTs/GCE (**b**) and MWNTs-IL/GCE (**c**).

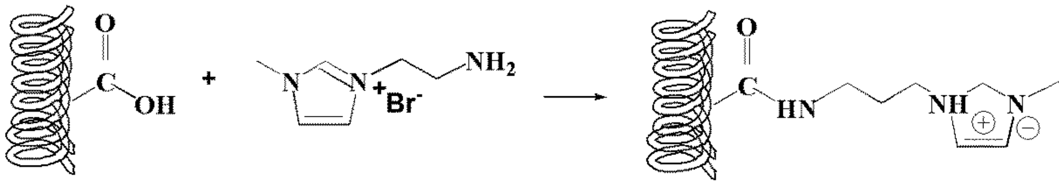

Figure S2. Scheme of the reaction for amine-terminated IL and functionalized MWNT.

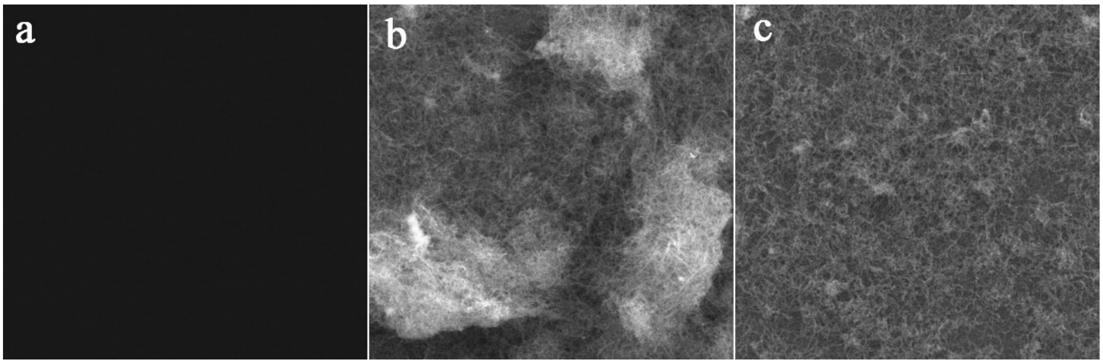

Figure S3. SEM images of bare GCE (**a**), MWNTs/GCE (**b**) and MWNTs-IL/GCE (**c**).
